# Case Report: Metabolic concordance and discordance in oligolesional disease—challenges in interpreting concurrent thyroid incidentaloma and vertebral lesions on ^18^F-FDG PET/CT

**DOI:** 10.3389/fmed.2026.1820027

**Published:** 2026-04-07

**Authors:** Xun Wang, Huizhen Xi, Xiao Zhi, Jundong Yang, Ming Gao, Guqing Zhang, Shuang Ge

**Affiliations:** 1 Department of Medical Imaging, Affiliated Hospital of Jining Medical University, Jining, China; 2 Department of Radiation Oncology, Affiliated Hospital of Jining Medical University, Jining, Shandong, China; 3 Department of Pathology, Affiliated Hospital of Jining Medical University, Jining, China

**Keywords:** Langerhans cell histiocytosis, metabolic pattern, PET/CT, thyroid incidentaloma, vertebral metastasis

## Abstract

**Background:**

2-deoxy-2-[18F]fluoro-D-glucose (^18^F-FDG) positron emission tomography/computed tomography (PET/CT) plays a pivotal role in the evaluation of vertebral lesions and systemic disease assessment. When vertebral lesions and thyroid incidentaloma are detected simultaneously, determining their relationship can be challenging. Metabolic similarity between lesions is often considered suggestive of a common origin, such as a primary tumor with its metastasis, whereas marked metabolic disparity may suggest separate primary tumors. However, both assumptions may be misleading.

**Case:**

We report two cases demonstrating opposite diagnostic pitfalls related to ^18^F-FDG metabolic patterns. In the first case, a mildly ^18^F-FDG-avid thyroid incidentaloma accompanied by a markedly hypermetabolic vertebral lesion raised suspicion for an unrelated primary bone tumor, such as plasmacytoma. Histopathological examination, however, confirmed follicular thyroid carcinoma with vertebral metastasis, illustrating a misleading metabolic discordance. In contrast, the second case showed comparable ^18^F-FDG uptake in both thyroid incidentaloma and vertebral lesion, initially suggesting a metastasis of thyroid carcinoma to the vertebrae. Surprisingly, the vertebral lesion was pathologically proven to be Langerhans cell histiocytosis (LCH) in a patient with a follicular thyroid neoplasm, representing a deceptive metabolic concordance.

**Conclusion:**

These cases highlight that both discordant and concordant ^18^F-FDG uptake patterns on PET/CT may lead to diagnostic pitfalls when used as the sole criterion for determining the relationship between coexisting lesions. Careful integration of metabolic findings with morphologic imaging features and histopathological confirmation is essential. When interpreted in appropriate clinical context, PET/CT remains invaluable for whole-body assessment, therapeutic planning, and individualized management.

## Introduction

2-deoxy-2-[18F]fluoro-D-glucose (^18^F-FDG) positron emission tomography/computed tomography (PET/CT) is widely used in the assessment of vertebral lesions, particularly in elderly patients with known or suspected malignancies, for whom bone metastasis is a major diagnostic concern. In routine clinical practice, vertebral lesions demonstrating ^18^F-FDG uptake comparable to that of a known primary tumor are usually interpreted as metastatic. This is in line with the general observation that ^18^F-FDG uptake in metastatic lesions tends to correlate with that of the primary tumor, whereas marked metabolic disparity may suggest a separate primary tumor (1, 2). However, reliance solely on metabolic comparisons can be misleading and result in diagnostic pitfalls.

Thyroid incidentaloma is defined as incidentally detected thyroid ^18^F-FDG uptake on imaging performed for a nonthyroid-related purpose; its nature often cannot be determined without further evaluation (3). The incidence of thyroid incidentalomas detected by ^18^F-FDG PET/CT is generally low, reported in less than 3% of all scans; however, focal ^18^F-FDG-avid lesions have a significant risk of malignancy (about one-third), and warrant further evaluation with ultrasound-guided fine-needle aspiration cytology (FNAC) (3, 4). When vertebral lesions and a thyroid incidentaloma are identified concurrently on ^18^F-FDG PET/CT, determining their relationship can be particularly challenging.

Here, we report two cases with coexisting vertebral lesions and thyroid incidentaloma, illustrating opposite but equally misleading PET/CT metabolic patterns. Although metabolic activity alone may be insufficient for accurate diagnosis, PET/CT remains valuable for whole-body assessment, treatment strategy selection, and prognostic evaluation. Therefore, metabolic findings should be interpreted alongside morphologic imaging features and confirmed by histopathology to ensure accurate diagnosis and appropriate management.

## Case presentation

### Case 1

**Figure 1:**
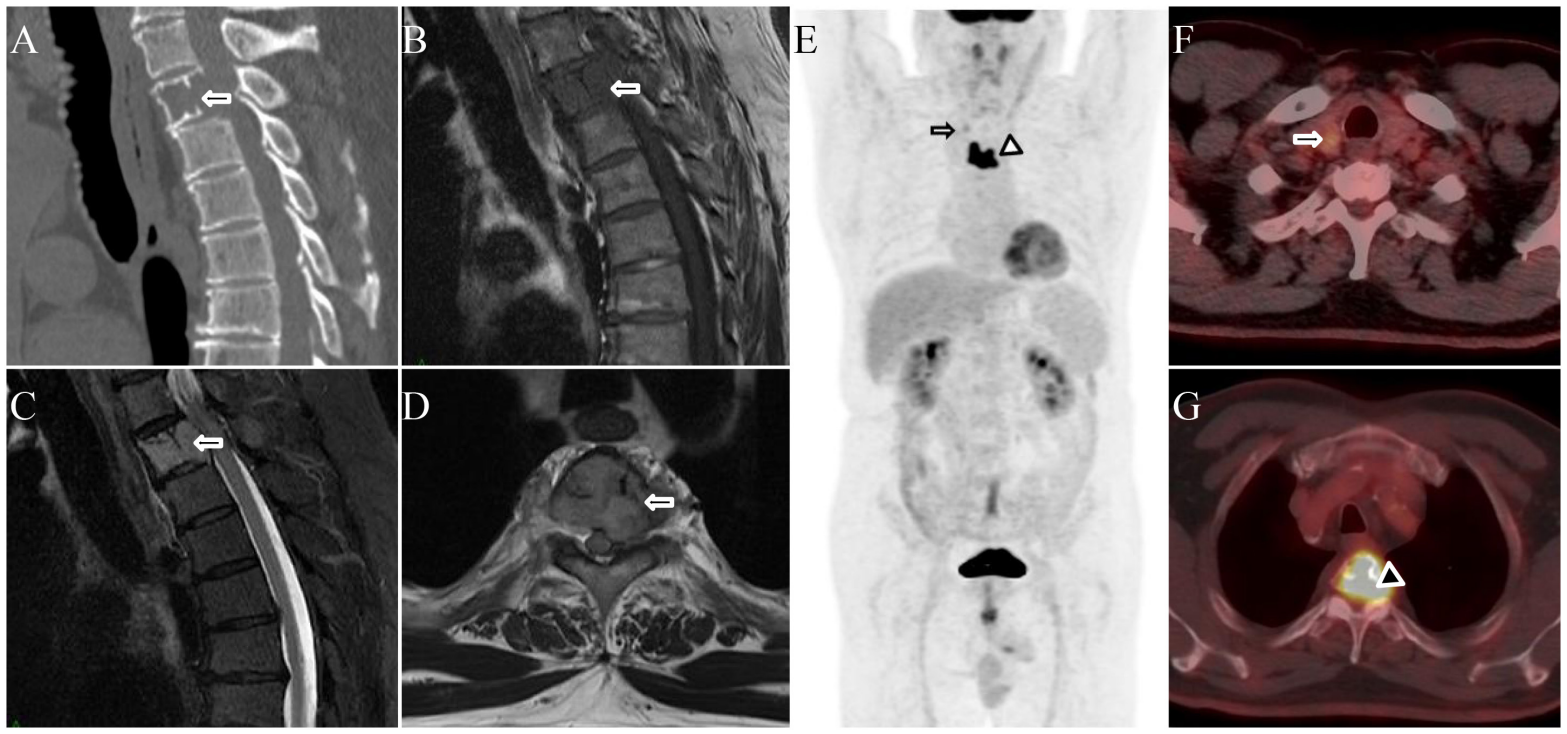
**(A)** CT showing osteolytic lesions involving the T3 vertebral body and bilateral pedicles, with residual bony ridges. **(B)** T1-weighted MRI showing hypointense signal in the T3 vertebral body. **(C)** Fat-suppressed T2-weighted MRI showing hyperintense signal in the same region. **(D)** Axial MRI demonstrating the characteristic “mini-brain”–like appearance, with narrowing of the adjacent spinal canal and left intervertebral foramen, resulting in compression of the left nerve root. **(E)** Maximum-intensity projection (MIP) 18F-FDG PET image showing intense uptake in the T3 vertebra (arrowhead) and mild uptake in the right thyroid lobe (arrow). **(F)** Axial PET/CT demonstrating a hypodense lesion with punctate calcifications in the right thyroid lobe (SUVmax 3.1). **(G)** Axial PET/CT showing osteolytic destruction of the T3 vertebra with posterior soft-tissue extension into the spinal canal, and markedly increased FDG uptake (SUVmax 15.2).

A 64-year-old man presented with a 2-month history of back pain without an identifiable precipitating factor. The pain was aggravated by physical activity and changes in posture. He had a 13-year history of hypertension and denied trauma, prior malignancy, or relevant family history. Physical examination revealed localized tenderness over the thoracic spine, without obvious spinal deformity or neurological deficits. The pain intensity was rated 3–5 on a Visual Analog Scale (VAS). Routine laboratory investigations revealed normal complete blood count, hepatic and renal function parameters, as well as serum tumor markers, including carcinoembryonic antigen (CEA), carbohydrate antigen 19-9 (CA19-9), prostate-specific antigen (PSA), and alpha-fetoprotein (AFP). CT demonstrated osteolytic destruction of the T3 vertebral body and bilateral pedicles, with residual bony ridges visible on sagittal images (Figure 1A). MRI showed hypointense signal on T1-weighted images (Figure 1B) and hyperintense signal on fat-suppressed T2-weighted images (Figure 1C), with a characteristic “mini-brain”–like appearance on axial images, accompanied by narrowing of the adjacent spinal canal and left intervertebral foramen, with compression of the left nerve root (Figure 1D). To further characterize the vertebral lesion and assess systemic involvement, whole-body ^18^F-FDG PET/CT examination was performed on May 4, 2022. Maximum-intensity projection (MIP) PET image (Figure 1E) showed intense ^18^F-FDG uptake in the T3 vertebra (arrowhead) and mild uptake in the right thyroid lobe (arrow). Axial fused PET/CT revealed a hypodense lesion with punctate calcifications in the right thyroid lobe, showing a maximum standardized uptake value (SUVmax) of 3.1 (Figure 1F). The T3 vertebral lesion showed osteolytic destruction with posterior soft-tissue extension into the spinal canal and markedly increased ^18^F-FDG uptake (SUVmax 15.2; Figure 1G). Based on imaging, the vertebral lesion was initially suspected to be solitary plasmacytoma (SP), whereas the thyroid incidentaloma was considered indeterminate, warranting further evaluation by thyroid ultrasound and FNAC. Surgical resection of the T3 vertebral lesion was performed on May 7, 2022, to alleviate symptoms and prevent potential neurological compromise. Histopathology revealed clusters of atypical cells infiltrating bone and adjacent soft tissue. Immunohistochemistry showed CK(+), TG(+), TTF-1(+), Vimentin(+), CK7(+), CK19(-), Galactin3(+), NapsinA(-), Pax-8(+), Ki-67(+, < 10%), raising suspicion for metastatic follicular thyroid carcinoma. FNAC of the thyroid incidentaloma suggested a follicular neoplasm (Bethesda category IV). Total thyroidectomy was performed on May 27, 2022 confirming follicular thyroid carcinoma. Postoperatively, the patient received radioactive iodine (^131^I, 150mCi), and zoledronic acid for bone repair. Despite treatment, serum thyroglobulin remained elevated (Tg >500 ng/ml), indicating poor therapeutic response. In January 2026, recurrent back pain (VAS 1–5) developed, and CT revealed new osteolytic lesions in T1–T4 with a soft tissue mass, suggesting tumor recurrence. A second course of ^131^I therapy (200 mCi) was administered on February 27, 2026.

### Case 2

**Figure 2:**
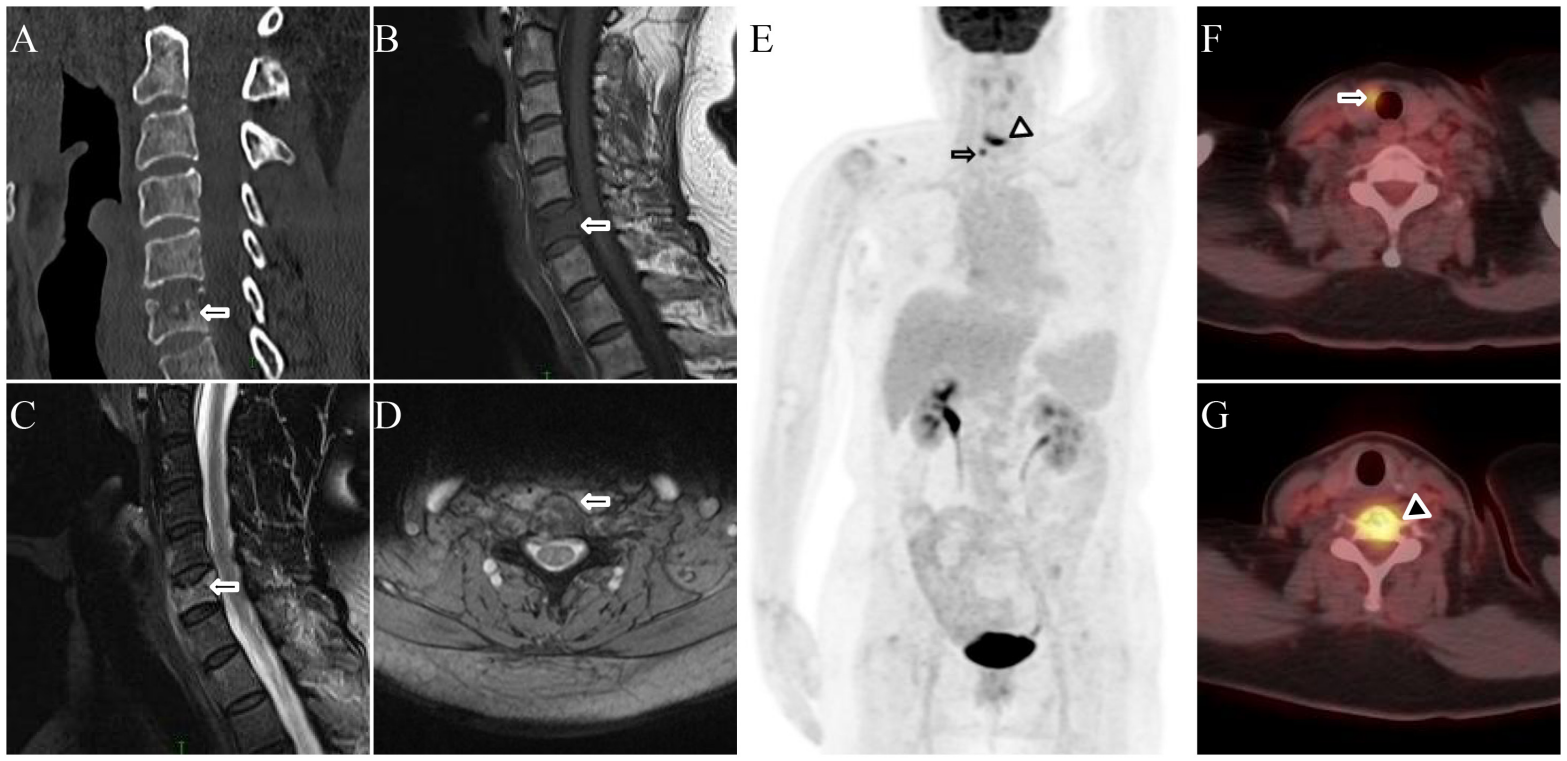
**(A)** CT showing a hypodense lesion with compression fracture in the C6 vertebral body. **(B)** T1-weighted MRI demonstrating hypointense signal in the C6 vertebra. **(C)** Fat-suppressed T2-weighted MRI showing hyperintense signal in the same region. **(D)** Axial MRI revealing surrounding soft-tissue swelling and edema. **(E)** Maximum-intensity projection (MIP) PET image showing 18F-FDG-avid lesions in the C6 vertebra (arrowhead) and right thyroid lobe (arrow). **(F)** Axial PET/CT demonstrating a hypodense lesion with ill-defined margins in the right thyroid lobe (SUVmax 6.1). **(G)** Axial PET/CT showing intravertebral hypodensity in the C6 vertebra with markedly increased FDG uptake (SUVmax 10.8).

A 71-year-old woman presented with a 2-week history of neck and upper back pain without an identifiable precipitating factor, accompanied by marked limitation of cervical motion. She had a history of hypertension and diabetes mellitus, both well controlled with medication. On October 16, 2023, she suffered a right humeral fracture after a fall and underwent internal fixation. She denied any prior history of malignancy or relevant family history of hereditary disease. Physical examination revealed tenderness over the cervical and upper thoracic vertebrae, with limited cervical range of motion but no apparent neurological deficits. The intensity of pain was rated 2–5 on the VAS. Routine laboratory investigations revealed normal complete blood count, hepatic and renal function, and serum tumor marker levels, including CEA, CA19-9, carbohydrate antigen 125 (CA125), carbohydrate antigen 15-3 (CA15-3) and AFP. CT demonstrated a hypodense lesion with compression fracture in the C6 vertebral body (Figure 2A). MRI revealed vertebral flattening with hypointense signal on T1-weighted images (Figure 2B) and hyperintense signal on fat-suppressed T2-weighted images (Figure 2C), accompanied by surrounding soft-tissue swelling and edema (Figure 2D). For comprehensive assessment of the vertebral lesion and systemic disease involvement, whole-body ^18^F-FDG PET/CT examination was performed on May 27, 2024. MIP PET image (Figure 2E) showed ^18^F-FDG-avid lesions in the C6 vertebra (arrowhead) and right thyroid lobe (arrow). On axial fused PET/CT, a hypodense lesion with ill-defined margins in the right thyroid lobe showed increased ^18^F-FDG uptake (SUVmax 6.1; Figure 2F), whereas the C6 vertebral body demonstrated compression deformity with associated flattening, intravertebral hypodensity and increased ^18^F-FDG uptake (SUVmax 10.8; Figure 2G). Thyroid carcinoma with vertebral metastasis was initially suspected given the comparable levels of metabolic activity between the two lesions. Ultrasound-guided FNAC of the thyroid incidentaloma, performed on May 31, 2024, was classified as Bethesda category IV (follicular neoplasm). Subsequently, surgical resection of the vertebral lesion was performed on June 13, 2024 to improve functional mobility and alleviate symptoms. Histopathology confirmed Langerhans cell histiocytosis (LCH), with immunohistochemistry showing CK (–), TG (–), CD68 (+), S-100 (+), CD1a (+), CD207 (+), ALK (–), and Ki-67 (+, 30%). Postoperatively, recovery from the vertebral lesion was uneventful, with marked alleviation of back pain and no procedure-related complications. No additional treatment was administered for the vertebral lesion. However, the patient declined further diagnostic evaluation and surgical treatment of the thyroid incidentaloma. On telephone follow-up to date, the patient remains in good general condition without significant discomfort.

## Discussion

^18^F-FDG PET/CT plays a crucial role in oncologic imaging, enabling a comprehensive assessment of tumor metabolism, lesion detection, staging, and treatment response throughout the body. Previous studies have suggested that, in lung cancer, the SUVs of most metastatic lesions fall within approximately 50%−200% of the primary tumor value, with deviations partly influenced by lesion size, and values outside this range should prompt consideration of alternative diagnoses (2, 5). However, SUV values may be influenced by several factors, including tumor differentiation, lesion size, and partial-volume effects, particularly in small lesions where ^18^F-FDG uptake may be underestimated due to the limited spatial resolution of PET imaging (2, 6). Therefore, reliance solely on the degree of ^18^F-FDG uptake for definitive diagnosis may be misleading. The present cases illustrate two contrasting interpretative pitfalls that may arise when metabolic activity is used as the primary criterion for lesion characterization. In the first case, the primary follicular thyroid carcinoma demonstrated a relatively low SUVmax of 3.1, whereas the metastatic vertebral lesion exhibited a markedly elevated SUVmax of 15.2, far exceeding the expected 200% threshold. Conversely, in the second case, the metabolic activity of the thyroid incidentaloma and the vertebral lesion was comparable (SUVmax 6.1 vs. 10.8), initially raising suspicion of metastatic disease, but histopathology ultimately revealed two distinct and unrelated diseases.

Data on misleading metabolic concordance or discordance between coexisting ^18^F-FDG-avid lesions in PET/CT are limited, with most evidence derived from case reports or small series. Such phenomena have been reported relatively more frequently in lymphoma, for instance when coexisting with a second primary tumor or inflammatory lesions (7). Several reports in solid tumors have also described diagnostic pitfalls caused by metabolic concordance or discordance (1, 8, 9). Although these occurrences are uncommon, they underscore the importance of integrating PET/CT findings with clinical and histopathological information, particularly in patients with oligolesional disease. To our knowledge, this report uniquely illustrates two contrasting PET/CT interpretative pitfalls within the same clinical context of oligolesional disease and a PET-avid thyroid incidentaloma, demonstrating that both metabolic concordance and metabolic discordance between lesions may lead to diagnostic misinterpretation.

In the first case, the vertebral lesion was initially suspected to be SP, which typically presents as an isolated osteolytic lesion with or without soft-tissue extension and may demonstrate the characteristic “mini-brain” appearance in vertebral lesions (10, 11). On conventional MRI, bone plasmacytomas show less peritumoral edema, more frequent T1 hyperintensity, and more homogeneous T2 and contrast-enhanced T1 signal compared with bone metastases. Diffusion-weighted imaging (DWI) further aids differentiation, with plasmacytomas demonstrating lower mean apparent diffusion coefficient (ADC) values, and an ADC ≤ 908 mm^2^/s yielding high sensitivity and specificity (12). In the second case, the vertebral lesion was initially suspected to represent metastasis from thyroid incidentaloma; however, histopathology revealed LCH. Acute-phase LCH typically presents as osteolytic lesions with map-like or moth-eaten bone destruction, sometimes with sclerotic margins and flattened vertebrae, accompanied by prominent surrounding edema, T1 hypointensity, T2 hyperintensity, and soft-tissue components on MRI (13). The ^18^F-FDG uptake activity of LCH bone lesions varies with disease activity, resulting in a wide range of SUVmax, reflecting metabolic heterogeneity on PET/CT (14–16). However, ^18^F-FDG PET/CT plays a crucial role in evaluating bone involvement and may be superior to conventional imaging techniques, facilitating the detection and characterization of bone lesions, thereby assisting in differential diagnosis and improving diagnostic accuracy (16).

Distant metastases from follicular thyroid carcinoma most commonly involve the lungs and bones (17). Previous studies have shown that the timing of metastatic presentation is not the key determinant of survival; instead, loss of radioiodine uptake serves as a more important prognostic factor, reflecting tumor dedifferentiation, as demonstrated by Albano et al. (18). This dedifferentiation is often associated with increased ^18^F-FDG uptake. Lesions with increased ^18^F-FDG avidity and/or loss of radioiodine uptake may exhibit the “flipflop phenomenon”, indicating dedifferentiation, more aggressive biology, and poorer prognosis, whereas low or absent ^18^F-FDG uptake with preserved ^131^I avidity suggests well-differentiated tumor tissue (6, 18, 19). In the first case, the markedly elevated SUVmax of the vertebral lesion suggested tumor dedifferentiation, which was consistent with the unfavorable clinical outcome despite surgery and subsequent ^131^I therapy. Thus, ^18^F-FDG PET may serve as a functional biomarker of tumor differentiation and prognosis in recurrent or metastatic thyroid carcinoma.

The role of PET/CT extends beyond lesion detection and prognostication to guiding treatment strategy and clinical management. In the first case, PET/CT identified a solitary bone metastasis in addition to the primary thyroid carcinoma. Previous studies have demonstrated that local consolidative therapy, including radiotherapy or surgery, is effective in patients with oligometastatic disease and can improve progression-free and overall survival compared with systemic therapy alone (20, 21). Accordingly, the patient underwent surgical resection of both the primary thyroid carcinoma and the metastatic bone lesion, followed by adjuvant radioiodine therapy as systemic treatment. In the second case, PET/CT delineated two lesions of distinct etiologies—a follicular thyroid neoplasm in the thyroid gland and vertebral involvement by LCH—with whole-body PET/CT demonstrating no evidence of additional LCH lesions. This effectively excluded multifocal disease that would have necessitated systemic therapy. Given that unifocal LCH in adults is often curable and that local therapy alone may be sufficient in many cases according to current guidelines and consensus recommendations (22), the vertebral lesion was managed surgically, while unnecessary systemic treatment was avoided.

Clinically, for patients with concurrent PET-avid thyroid incidentaloma and vertebral lesions, a stepwise approach is recommended. First, perform a comprehensive clinical and laboratory evaluation and carefully review imaging features to identify any suspicious conditions. Second, interpret PET/CT metabolic patterns cautiously, recognizing that both concordance and discordance between lesions can be misleading, and avoid relying solely on SUV values to infer their relationship. Third, obtain targeted histopathological confirmation, including ultrasound-guided FNAC of the thyroid incidentaloma and, if indicated, biopsy of the vertebral lesion. Finally, management decisions should be guided by multidisciplinary consultation involving endocrinology, nuclear medicine, radiology, oncology, and surgery. This framework emphasizes that integrated assessment is essential, particularly in patients with oligolesional disease, to reduce diagnostic misinterpretation and optimize clinical decision-making.

## Conclusion

In these two cases, ^18^F-FDG PET/CT highlighted the diagnostic and management challenges posed by coexisting thyroid incidentaloma and vertebral lesions, especially in patients presenting with oligolesional disease. Although PET/CT provides valuable whole-body metabolic information, ^18^F-FDG uptake intensity alone is insufficient to determine lesion origin or biological aggressiveness. Substantial metabolic heterogeneity may exist between primary and metastatic lesions, and distinct diseases can exhibit overlapping imaging features. In the setting of oligolesional disease, histopathological confirmation of the critical lesion(s) is essential for therapeutic planning, especially when treatment strategies differ substantially. Accurate interpretation of PET/CT findings, integrated with pathology and clinical context, is crucial for avoiding misdiagnosis, inappropriate attribution of metastasis, and unnecessary systemic treatment. When applied judiciously, PET/CT can refine differential diagnosis and guide individualized management, including the selection of local therapy in patients with oligometastatic or unifocal disease.

## Data Availability

The original contributions presented in the study are included in the article/supplementary material, further inquiries can be directed to the corresponding authors.
